# *N*-(4-Chloro­phen­yl)-3-methyl­benzamide

**DOI:** 10.1107/S1600536809041956

**Published:** 2009-10-17

**Authors:** B. Thimme Gowda, Miroslav Tokarčík, Jozef Kožíšek, Vinola Zeena Rodrigues, Hartmut Fuess

**Affiliations:** a Department of Chemistry, Mangalore University, Mangalagangotri 574 199, Mangalore, India; b Faculty of Chemical and Food Technology, Slovak Technical University, Radlinského 9, SK-812 37 Bratislava, Slovak Republic; c Institute of Materials Science, Darmstadt University of Technology, Petersenstrasse 23, D-64287, Darmstadt, Germany

## Abstract

In the structure of the title compound, C_14_H_12_ClNO, the conformations of the N—H and C=O bonds are *anti* to each other. Furthermore, the conformation of the C=O bond is *syn* to the *meta*-methyl group in the benzoyl ring. The central –NH—C(=O)– amido group makes a dihedral angle of 32.4 (1)° with the benzoyl ring and 36.1 (1)° with the anilino ring. The dihedral angle between the two benzene rings is 68.4 (1)°. In the crystal, inter­molecular N—H⋯O hydrogen bonds link the mol­ecules into chains running along the *a* axis

## Related literature

For the preparation of the title compound, see: Gowda *et al.* (2003 ▶). For related structures, see: Bowes *et al.* (2003 ▶); Gowda, Foro *et al.* (2008 ▶, 2009 ▶); Gowda, Tokarčík *et al.* (2008 ▶).
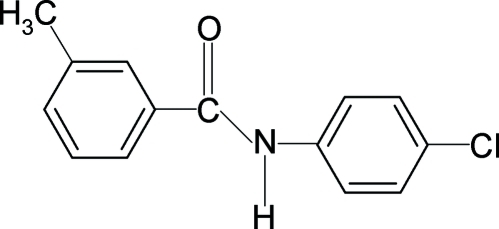


## Experimental

### 

#### Crystal data

C_14_H_12_ClNO
*M* *_r_* = 245.7
Monoclinic, 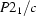

*a* = 5.31325 (9) Å
*b* = 13.9256 (2) Å
*c* = 16.3497 (3) Å
β = 93.1799 (16)°
*V* = 1207.86 (3) Å^3^
*Z* = 4
Mo *K*α radiation
μ = 0.30 mm^−1^
*T* = 295 K
0.54 × 0.41 × 0.24 mm

#### Data collection

Oxford Diffraction Xcalibur, Ruby, Gemini diffractometer
Absorption correction: analytical (CrysAlisPro; Oxford Diffraction, 2009 ▶) *T*_min_ = 0.842, *T*_max_ = 0.933
22561 measured reflections
2327 independent reflections
2083 reflections with *I* > 2σ(*I*)
*R*_int_ = 0.021

#### Refinement

*R*[*F* ^2^ > 2σ(*F* ^2^)] = 0.034
*wR*(*F* ^2^) = 0.093
*S* = 1.08
2327 reflections
160 parameters
1 restraint
H atoms treated by a mixture of independent and constrained refinement
Δρ_max_ = 0.20 e Å^−3^
Δρ_min_ = −0.25 e Å^−3^

### 

Data collection: *CrysAlisPro* (Oxford Diffraction, 2009 ▶); cell refinement: *CrysAlisPro*; data reduction: *CrysAlisPro*; program(s) used to solve structure: *SHELXS97* (Sheldrick, 2008 ▶); program(s) used to refine structure: *SHELXL97* (Sheldrick, 2008 ▶); molecular graphics: *ORTEP-3* (Farrugia, 1997 ▶) and *DIAMOND* (Brandenburg, 2002 ▶); software used to prepare material for publication: *SHELXL97*, *PLATON* (Spek, 2009 ▶) and *WinGX* (Farrugia, 1999 ▶).

## Supplementary Material

Crystal structure: contains datablocks I. DOI: 10.1107/S1600536809041956/om2285sup1.cif

Structure factors: contains datablocks I. DOI: 10.1107/S1600536809041956/om2285Isup2.hkl

Additional supplementary materials:  crystallographic information; 3D view; checkCIF report

## Figures and Tables

**Table 1:** Hydrogen-bond geometry (Å, °)

*D*—H⋯*A*	*D*—H	H⋯*A*	*D*⋯*A*	*D*—H⋯*A*
N1—H1*N*⋯O1^i^	0.864 (14)	2.298 (14)	3.1019 (16)	154.7 (15)

Symmetry code: (i) 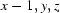.

**Fig. 1.**
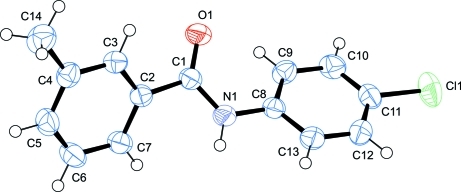
Molecular structure of (I) showing the atom labelling scheme. Displacement ellipsoids are drawn at the 50% probability level. H atoms are represented as small spheres of arbitrary radii.

**Fig. 2.**
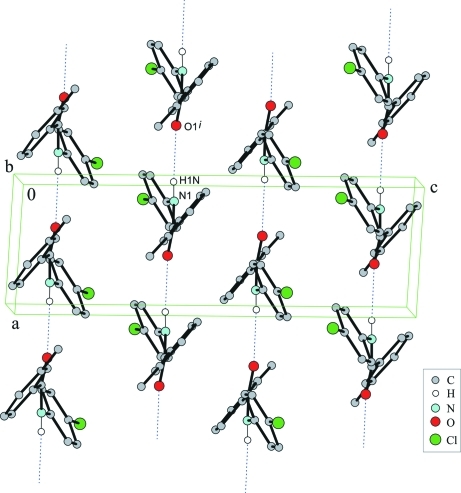
Molecular packing of (I) with N–H···O hydrogen bonds shown as dashed lines. Symmetry code (i): x - 1,y,z. H atoms not involved in hydrogen bonding have been omitted.

## supplementary crystallographic information

### Comment

In the present work, as part of a study of the substituent effects on the solid state structures of benzanilides (Gowda, Foro *et al.*, 2008, 2009; Gowda, Tokarčík *et al.*, 2008), the structure of *N*-(4-chlorophenyl)3-methylbenzamide (I) has been determined. In the structure, the conformations of the N—H and C=O bonds are *anti* to each other (Fig. 1), similar to those observed in *N*-(4-chlorophenyl)2-methylbenzamide (II)(Gowda, Foro *et al.*, 2008), *N*-(4-chlorophenyl)benzamide (III)(Gowda, Tokarčík *et al.*, 2008), 3-methyl-*N*-(phenyl)benzamide (IV) (Gowda, Foro *et al.*, 2008) and the parent benzanilide (Bowes *et al.*, 2003). Further, the conformation of the C=O bond in (I) is *syn* to the *meta*-methyl substituent in the benzoyl ring. The central amido group –NH—C(=O)– makes a dihedral angle of 32.4 (1)° with the methyl-phenyl ring (benzoyl) and 36.1 (1)° with the chloro-phenyl ring (anilino). The dihedral angle between the two benzene rings is 68.4 (1)°, compared to the values of 83.1 (1)° in (II), 60.76 (3)°) in (III), and 22.17 (18)° & 75.86 (12)°, respectively, in molecules 1 and 2 of (IV).

The packing diagram of molecules in (I) showing the intermolecular N–H···O hydrogen bonds (Table 1) involved in the formation of molecular chains running along the *a*-axis is shown in Fig. 2.

### Experimental

The title compound was prepared according to the literature method (Gowda *et al.*, 2003). The purity of the compound was checked by determining its melting point. It was characterized by recording its infrared and NMR spectra. Single crystals of the title compound used in X-ray diffraction studies were obtained from a slow evaporation of its ethanolic solution at room temperature.

### Refinement

All hydrogen atoms were seen in difference map. H atom attached to nitrogen was refined with the N—H distance restrained to 0.86 (2) Å. H atoms attached to carbon atoms were placed in calculated positions with C–H distances of 0.93 Å (C aromatic) and 0.96 Å (C methyl). The *U*_iso_(H) values were set at 1.2*U*_eq_(C aromatic, N) and 1.5 *U*_eq_(C methyl). The C14 methyl group shows orientational disorder in the hydrogen atom positions. The two sets of methyl hydrogen atoms were refined with equal occupancy.

### Crystal data

**Table:**

C_14_H_12_ClNO	*F*(000) = 512
*M**_r_* = 245.7	*D*_x_ = 1.351 Mg m^−^^3^
Monoclinic, *P*2_1_/*c*	Mo *K*α radiation, λ = 0.71073 Å
Hall symbol: -P 2ybc	Cell parameters from 15854 reflections
*a* = 5.31325 (9) Å	θ = 2.5–29.5°
*b* = 13.9256 (2) Å	µ = 0.30 mm^−^^1^
*c* = 16.3497 (3) Å	*T* = 295 K
β = 93.1799 (16)°	Block, colourless
*V* = 1207.86 (3) Å^3^	0.54 × 0.41 × 0.24 mm
*Z* = 4	

### Data collection

**Table:**

Oxford Diffraction Xcalibur, Ruby, Gemini diffractometer	2327 independent reflections
graphite	2083 reflections with *I* > 2σ(*I*)
Detector resolution: 10.434 pixels mm^-1^	*R*_int_ = 0.021
ω scans	θ_max_ = 25.8°, θ_min_ = 2.5°
Absorption correction: analytical (*CrysAlis PRO*; Oxford Diffraction, 2009)	*h* = −6→6
*T*_min_ = 0.842, *T*_max_ = 0.933	*k* = −17→17
22561 measured reflections	*l* = −19→19

### Refinement

**Table:**

Refinement on *F*^2^	Secondary atom site location: difference Fourier map
Least-squares matrix: full	Hydrogen site location: inferred from neighbouring sites
*R*[*F*^2^ > 2σ(*F*^2^)] = 0.034	H atoms treated by a mixture of independent and constrained refinement
*wR*(*F*^2^) = 0.093	*w* = 1/[σ^2^(*F*_o_^2^) + (0.0428*P*)^2^ + 0.3304*P*] where *P* = (*F*_o_^2^ + 2*F*_c_^2^)/3
*S* = 1.08	(Δ/σ)_max_ = 0.001
2327 reflections	Δρ_max_ = 0.20 e Å^−^^3^
160 parameters	Δρ_min_ = −0.25 e Å^−^^3^
1 restraint	Extinction correction: *SHELXL*, Fc^*^=kFc[1+0.001xFc^2^λ^3^/sin(2θ)]^-1/4^
Primary atom site location: structure-invariant direct methods	Extinction coefficient: 0.0092 (14)

### Special details

**Table:**

Geometry. All e.s.d.'s (except the e.s.d. in the dihedral angle between two l.s. planes) are estimated using the full covariance matrix. The cell e.s.d.'s are taken into account individually in the estimation of e.s.d.'s in distances, angles and torsion angles; correlations between e.s.d.'s in cell parameters are only used when they are defined by crystal symmetry. An approximate (isotropic) treatment of cell e.s.d.'s is used for estimating e.s.d.'s involving l.s. planes.
Refinement. Refinement of *F*^2^ against ALL reflections. The weighted *R*-factor *wR* and goodness of fit *S* are based on *F*^2^, conventional *R*-factors *R* are based on *F*, with *F* set to zero for negative *F*^2^. The threshold expression of *F*^2^ > σ(*F*^2^) is used only for calculating *R*-factors(gt) *etc*. and is not relevant to the choice of reflections for refinement. *R*-factors based on *F*^2^ are statistically about twice as large as those based on *F*, and *R*- factors based on ALL data will be even larger.

### Fractional atomic coordinates and isotropic or equivalent isotropic displacement parameters (Å^2^)

**Table:**

	*x*	*y*	*z*	*U*_iso_*/*U*_eq_	Occ. (<1)
N1	0.1646 (2)	0.45549 (8)	0.39171 (8)	0.0412 (3)	
H1N	0.017 (3)	0.4290 (12)	0.3885 (10)	0.049*	
O1	0.58424 (19)	0.42883 (8)	0.38537 (8)	0.0557 (3)	
C1	0.3719 (2)	0.39844 (10)	0.39515 (8)	0.0386 (3)	
C2	0.3269 (2)	0.29460 (10)	0.41211 (8)	0.0369 (3)	
C3	0.4867 (3)	0.22789 (10)	0.37862 (9)	0.0405 (3)	
H3	0.6157	0.2493	0.347	0.049*	
C4	0.4574 (3)	0.13000 (10)	0.39146 (9)	0.0431 (3)	
C5	0.2706 (3)	0.10059 (11)	0.44192 (10)	0.0498 (4)	
H5	0.2506	0.0355	0.4525	0.06*	
C6	0.1136 (3)	0.16637 (12)	0.47667 (10)	0.0522 (4)	
H6	−0.0093	0.1451	0.5108	0.063*	
C7	0.1374 (3)	0.26343 (11)	0.46128 (9)	0.0437 (3)	
H7	0.0283	0.3073	0.4835	0.052*	
C8	0.1610 (2)	0.55455 (10)	0.37157 (8)	0.0366 (3)	
C9	0.3518 (3)	0.61698 (11)	0.39831 (9)	0.0443 (3)	
H9	0.4893	0.5941	0.4302	0.053*	
C10	0.3380 (3)	0.71322 (11)	0.37762 (10)	0.0482 (4)	
H10	0.4658	0.7551	0.3955	0.058*	
C11	0.1347 (3)	0.74660 (11)	0.33054 (9)	0.0447 (4)	
C12	−0.0573 (3)	0.68588 (11)	0.30389 (10)	0.0489 (4)	
H12	−0.1946	0.7093	0.2722	0.059*	
C13	−0.0436 (3)	0.58985 (11)	0.32472 (9)	0.0449 (3)	
H13	−0.173	0.5485	0.3071	0.054*	
C14	0.6205 (3)	0.05784 (13)	0.35038 (12)	0.0605 (4)	
H14A	0.7546	0.0907	0.325	0.091*	0.5
H14B	0.5204	0.0233	0.3095	0.091*	0.5
H14C	0.6904	0.0136	0.3905	0.091*	0.5
H14D	0.5557	−0.0056	0.3583	0.091*	0.5
H14E	0.7899	0.0617	0.3738	0.091*	0.5
H14F	0.6198	0.0715	0.2928	0.091*	0.5
Cl1	0.11527 (11)	0.86780 (3)	0.30416 (3)	0.07259 (19)	

### Atomic displacement parameters (Å^2^)

**Table:**

	*U*^11^	*U*^22^	*U*^33^	*U*^12^	*U*^13^	*U*^23^
N1	0.0333 (6)	0.0357 (6)	0.0548 (7)	−0.0041 (5)	0.0028 (5)	0.0011 (5)
O1	0.0374 (5)	0.0426 (6)	0.0884 (9)	−0.0029 (4)	0.0141 (5)	0.0086 (6)
C1	0.0371 (7)	0.0378 (7)	0.0413 (7)	−0.0025 (6)	0.0052 (5)	0.0010 (6)
C2	0.0345 (6)	0.0381 (7)	0.0377 (7)	−0.0030 (5)	−0.0016 (5)	0.0008 (6)
C3	0.0355 (7)	0.0430 (8)	0.0429 (7)	−0.0015 (6)	0.0028 (6)	0.0010 (6)
C4	0.0418 (7)	0.0402 (8)	0.0465 (8)	0.0014 (6)	−0.0052 (6)	−0.0025 (6)
C5	0.0538 (9)	0.0363 (8)	0.0589 (9)	−0.0057 (7)	−0.0005 (7)	0.0045 (7)
C6	0.0524 (9)	0.0478 (9)	0.0577 (9)	−0.0102 (7)	0.0146 (7)	0.0050 (7)
C7	0.0409 (7)	0.0420 (8)	0.0490 (8)	−0.0025 (6)	0.0093 (6)	−0.0008 (6)
C8	0.0355 (7)	0.0354 (7)	0.0393 (7)	−0.0015 (5)	0.0068 (5)	−0.0012 (5)
C9	0.0390 (7)	0.0420 (8)	0.0511 (8)	0.0002 (6)	−0.0043 (6)	−0.0048 (6)
C10	0.0449 (8)	0.0397 (8)	0.0600 (9)	−0.0089 (6)	0.0028 (7)	−0.0082 (7)
C11	0.0532 (9)	0.0361 (7)	0.0460 (8)	−0.0013 (6)	0.0133 (7)	0.0022 (6)
C12	0.0464 (8)	0.0474 (8)	0.0523 (9)	0.0027 (7)	−0.0026 (7)	0.0074 (7)
C13	0.0373 (7)	0.0428 (8)	0.0540 (9)	−0.0059 (6)	−0.0019 (6)	0.0007 (7)
C14	0.0588 (10)	0.0486 (9)	0.0743 (12)	0.0070 (8)	0.0055 (8)	−0.0091 (8)
Cl1	0.0979 (4)	0.0399 (2)	0.0805 (3)	−0.0026 (2)	0.0090 (3)	0.0145 (2)

### Geometric parameters (Å, °)

**Table:**

N1—C1	1.3565 (18)	C8—C9	1.388 (2)
N1—C8	1.4181 (18)	C9—C10	1.383 (2)
N1—H1N	0.864 (14)	C9—H9	0.93
O1—C1	1.2239 (17)	C10—C11	1.372 (2)
C1—C2	1.4942 (19)	C10—H10	0.93
C2—C3	1.391 (2)	C11—C12	1.377 (2)
C2—C7	1.3922 (19)	C11—Cl1	1.7437 (15)
C3—C4	1.389 (2)	C12—C13	1.381 (2)
C3—H3	0.93	C12—H12	0.93
C4—C5	1.387 (2)	C13—H13	0.93
C4—C14	1.509 (2)	C14—H14A	0.96
C5—C6	1.382 (2)	C14—H14B	0.96
C5—H5	0.93	C14—H14C	0.96
C6—C7	1.382 (2)	C14—H14D	0.96
C6—H6	0.93	C14—H14E	0.96
C7—H7	0.93	C14—H14F	0.96
C8—C13	1.385 (2)		
			
C1—N1—C8	125.43 (12)	C9—C10—H10	120.2
C1—N1—H1N	118.9 (11)	C10—C11—C12	121.04 (14)
C8—N1—H1N	113.6 (11)	C10—C11—Cl1	120.05 (12)
O1—C1—N1	122.98 (13)	C12—C11—Cl1	118.91 (12)
O1—C1—C2	121.13 (12)	C11—C12—C13	119.25 (14)
N1—C1—C2	115.89 (12)	C11—C12—H12	120.4
C3—C2—C7	119.73 (13)	C13—C12—H12	120.4
C3—C2—C1	117.77 (12)	C12—C13—C8	120.61 (13)
C7—C2—C1	122.49 (13)	C12—C13—H13	119.7
C4—C3—C2	121.34 (13)	C8—C13—H13	119.7
C4—C3—H3	119.3	C4—C14—H14A	109.5
C2—C3—H3	119.3	C4—C14—H14B	109.5
C5—C4—C3	117.98 (14)	H14A—C14—H14B	109.5
C5—C4—C14	121.05 (14)	C4—C14—H14C	109.5
C3—C4—C14	120.96 (14)	H14A—C14—H14C	109.5
C6—C5—C4	121.12 (14)	H14B—C14—H14C	109.5
C6—C5—H5	119.4	C4—C14—H14D	109.5
C4—C5—H5	119.4	H14A—C14—H14D	141.1
C7—C6—C5	120.66 (14)	H14B—C14—H14D	56.3
C7—C6—H6	119.7	H14C—C14—H14D	56.3
C5—C6—H6	119.7	C4—C14—H14E	109.5
C6—C7—C2	119.11 (14)	H14A—C14—H14E	56.3
C6—C7—H7	120.4	H14B—C14—H14E	141.1
C2—C7—H7	120.4	H14C—C14—H14E	56.3
C13—C8—C9	119.29 (13)	H14D—C14—H14E	109.5
C13—C8—N1	118.28 (12)	C4—C14—H14F	109.5
C9—C8—N1	122.42 (13)	H14A—C14—H14F	56.3
C10—C9—C8	120.13 (14)	H14B—C14—H14F	56.3
C10—C9—H9	119.9	H14C—C14—H14F	141.1
C8—C9—H9	119.9	H14D—C14—H14F	109.5
C11—C10—C9	119.67 (13)	H14E—C14—H14F	109.5
C11—C10—H10	120.2		
			
C8—N1—C1—O1	4.3 (2)	C3—C2—C7—C6	0.7 (2)
C8—N1—C1—C2	−175.93 (12)	C1—C2—C7—C6	−177.55 (14)
O1—C1—C2—C3	−31.1 (2)	C1—N1—C8—C13	142.17 (15)
N1—C1—C2—C3	149.10 (13)	C1—N1—C8—C9	−39.0 (2)
O1—C1—C2—C7	147.24 (15)	C13—C8—C9—C10	−0.6 (2)
N1—C1—C2—C7	−32.57 (19)	N1—C8—C9—C10	−179.33 (14)
C7—C2—C3—C4	1.7 (2)	C8—C9—C10—C11	0.0 (2)
C1—C2—C3—C4	−179.96 (13)	C9—C10—C11—C12	0.5 (2)
C2—C3—C4—C5	−2.9 (2)	C9—C10—C11—Cl1	179.97 (12)
C2—C3—C4—C14	175.93 (14)	C10—C11—C12—C13	−0.3 (2)
C3—C4—C5—C6	1.7 (2)	Cl1—C11—C12—C13	−179.82 (12)
C14—C4—C5—C6	−177.08 (15)	C11—C12—C13—C8	−0.3 (2)
C4—C5—C6—C7	0.6 (3)	C9—C8—C13—C12	0.7 (2)
C5—C6—C7—C2	−1.9 (2)	N1—C8—C13—C12	179.54 (13)

### Hydrogen-bond geometry (Å, °)

**Table:**

*D*—H···*A*	*D*—H	H···*A*	*D*···*A*	*D*—H···*A*
N1—H1N···O1^i^	0.86 (1)	2.30 (1)	3.1019 (16)	155 (2)

Symmetry codes: (i) *x*−1, *y*, *z*.
